# Work engagement among health professionals in public health facilities of Bench-Sheko zone, southwest Ethiopia

**DOI:** 10.1186/s12913-023-09680-5

**Published:** 2023-06-27

**Authors:** Temesgen Ginbeto, Ayal Debie, Demiss Mulatu Geberu, Dereje Alemayehu, Endalkachew Dellie

**Affiliations:** 1 Department of Public Health, School of Public Health, College of Health Sciences, Mizan-Tepi University, Tepi, Ethiopia; 2 Department of Health Systems and Policy, Institute of Public Health, University of Gondar, Gondar, Ethiopia; 3 College of Medicine and Public Health, Flinders University, Adelaide, Australia

**Keywords:** Work engagement, Health professionals, Public health facilities, Southwest Ethiopia

## Abstract

**Background:**

The level of health professional work engagement affects retention, burnout, job satisfaction, patient satisfaction, and outcomes; however, there is a paucity of evidence that benefit health professional work engagement. Therefore, this study aimed to assess work engagement and associated factors among health professionals at public health facilities in the Bench-Sheko zone in southwest Ethiopia.

**Methods:**

Facility-based cross-sectional study was conducted among 605 health professionals from 29 March to 29 April 2021. A simple random sampling technique was used to select the participants. Data were collected using a self-administered questionnaire. Linear regression was fitted and those variables with p-value < 0.2 in simple linear regression were entered into multiple linear regression analysis. Unstandardized β-coefficient with 95% CI and p-value < 0.05 were used as the cut of points to determine the factors associated with work engagement.

**Results:**

Mean score percentage of work engagement was 71.8%. Health center staff (β = 0.31; 95% CI: 0.22, 0.40), married professionals (β = 0.10; 95% CI: 0.005, 0.17), co-worker support (β = 0.06; 95% CI: 0.004, 0.11), role clarity (β = 0.14; 95% CI: 0.07, 0.21), reward (β = 0.10; 95% CI: 0.05, 0.15), resilience (β = 0.14 95%; CI: 0.07, 0.21), self-efficacy (β = 0.24; 95% CI: 0.16, 0.31) and optimism (β = 0.20; 95% CI: 0.15, 0.26) were positively associated with work engagement. On the contrary, cognitive demand (β= -0.06; 95% CI: -0.11, -0.01) was negatively associated with work engagement.

**Conclusion:**

In this study, health professionals had a moderate level of work engagement. Health facilities shall improve their culture of co-worker support, role clarity, reward, resilience, self-efficacy, and optimism to enhance work engagement. Future researchers shall be done further studies to evaluate the relationship between cognitive demand and work engagement among health professionals.

**Supplementary Information:**

The online version contains supplementary material available at 10.1186/s12913-023-09680-5.

## Background

Human resource is essential for the success of any type of organization [1] and one of the most vital building blocks for a health system [2]. Availability of health workforce (HWF) in adequate numbers, skill mix, distribution, motivation, competence, and quality is very important for effective and equitable health service delivery, but all nations anywhere in the world face challenges on HWF in terms of their production, deployment, retention, and proper utilization [3, 4]. About 40% of health professionals (doctors, nurses, and midwives) would resign from their employment because of dissatisfaction with their work [5]. Ethiopia has also suffered from high geographic maldistribution, skills imbalance, poor retention, outmigration, and low productivity [6, 7]. Healthcare workers’ dissatisfaction with their professions was also another key problem in Ethiopia [8].

Work engagement is a positive, substantial, work-related mental state that includes vigor, dedication, and absorption [9]. It has a direct impact on the quality of care [10, 11]. Fully engaged employees have less job stress and depression than non-engaged employees [12] and a high level of work engagement can enhance job performance, job satisfaction, and emotional health and reduce turnover intention [13]. It also improves effectiveness, and patient satisfaction, reduces adverse events, and increases productivity [14, 15]. Only 13% of all worker groups [5] and about 27% of doctors and nurses worldwide are fully engaged in their work [16]. In Ethiopia, high turnover intention and burnout are currently common problems among health professionals [17, 18]. This can impede the delivery of primary healthcare services [19].

Work engagement is influenced by many factors, such as family [20], personality [21], exercise [22], and diet [23]. Reward, supervisor support, collaboration, and emotions can affect work engagement [24]. Organizational resources, such as autonomy, feedback, opportunities for professional training, and access to resources contribute to health professionals’ engagement [25, 26]. The respectful and mutually beneficial working relationship between employers and employees can enhance employee work engagement [27]. On the contrary, weak commitment, high dissatisfaction, high intent to leave, poor teamwork practice, high absenteeism, low productivity, deviant workplace behavior, high burnout, and poor innovation and creativity were the characteristics of non-engaged employees [28].

Ministry of Health - Ethiopia has been taking various measures to curb work engagement-related challenges by increasing the production of professionals, staffing, and expanding health facilities, and training institutes [6, 29]. However, there is a lack of evidence on the level and factors contributing to work engagement. This study therefore aimed to assess work engagement and associated factors among health professionals in public health facilities in southwest Ethiopia.

## Methods and materials

### Study design and settings

A facility-based cross-sectional study was conducted in the Bench Sheko zone, Southwest Ethiopia from 29 March to 29 April 2021. The zone has six districts and two town administrations with a total population of 625,345 and Mizan Aman is its capital town. Mizan Aman is located 561 km southwest of Addis Ababa (the Capital city of Ethiopia). The zone has 1 teaching hospital, 26 Health Centers (HCs), 9 medium clinics, 122 primary clinics, 35 private drug stores, and 128 health posts [30]. A total of 904 health professionals were working at the public health facilities of the Bench Sheko zone.

### Population

All health professionals working in the public health facilities of Bench Sheko zone were the source population while those health professionals who were permanently working in selected public health facilities of Bench Sheko zone were the study population. Health professionals in the selected health facilities who had less than six months of work experience in the study area were excluded from the study.

### Sample size and sampling procedure

The sample size was determined using single population proportion formula considering 50% of proportion (P) (since there were no previously published works on the subject), 95% confidence level, 5% margin of error, 1.5 design effect, and 5% of the non-response rate. That is, n = (Z_a/2_)^2^ *(P)(1-P)/(d)^2^; where “n” is the minimum possible sample size, “Z_α/2_” is the standard score value for 95% confidence level for two-sided normal distribution, “P” is the proportion of health workers work engagement, and “d” is margin of error. Thus, n = (1.96)^2^ (0.5*0.5)/ (0.05)^2^ = 384. At last, we multiplied our sample by 1.5 design effect and added a 5% non-response rate, and our final sample size was 605.

We first stratified the health facilities in the Bench-Sheko zone into hospitals and health centers. We then selected the hospital purposely since there was only one hospital (Mizan Aman Teaching University Hospital) in the zone and we selected 13 health centers (Mizan, Sheko, Eteka, Gizmeret, Debrework, Kite, Zozo, Gichi, Bebeka, Gedu, Bir, Gacheb, and Dizu) using lottery method. We had 334 and 339 health professionals included in one hospital and 13 selected health centers, respectively. We proportionally allocated our final sample size to each selected facility based on the number of health professionals after the exclusion of unselected health centers. We used a simple random sampling technique using their payroll list as a sampling frame to select the participants.

### Measurement of variables

Work engagement is a positive fulfillment of a work-related state of mind. It was measured by 17 Utrecht work engagement scale (UWES) items each containing a 5 -point Likert scale (1 = strongly disagree to 5 = strongly agree) [31] with a reliability test of Cronbach’s alpha (α = 0.94). The mean score was reported as the percentage of the mean score (% SM) and the value ranged from 0 to 100%. The %SM was calculated: %SM = (Actual score –P. Minimum score/P. Maximum – Minimum) *100 [31]. Level of work engagement was categorized based on the % SM as ≥ 95% “very high”; ≥ 75 to < 95% “high”; ≥ 25 to < 75% “average”; ≥ 5 to < 25% “low”; and < 5% “low” [31].

Work engagement was measured using three dimensions, including vigor (energy), dedication (involvement and significance), and absorption (concentration). Vigor is a high-level psychological energy and mental resilience during work. Dedication is the connection in someone’s work and experiencing a sense of significance, enthusiasm, and challenge. Absorption is being engrossed in and focused on someone’s work.

Supervisor support is the employee’s perception of the degree to which supervisors value their contributions and care about their well-being [32]. Coworker’s support is the extent to which individuals can count on information, assistance, and appreciation from their colleagues at work [32]. Cognitive demand is the level of thought and reasoning required to complete a task [32]. Emotional demand is the extent to which the job requires sustained emotional effort because of interactional contact with clients [32].

Role clarity is the degree to which an employee gets information about an expected task, results, and order of tasks to be done [32]. The reward is an incentive plan to reinforce the desired behavior of workers or employers in return for their service to the organization [32].

Resilience is a positive psychological capacity to rebound from adversity, uncertainty, conflict, failure, or even positive change [33]. The workload is the amount and pace of work to be performed [32]. Self-efficacy is people’s beliefs about their capabilities to produce designated levels of performance that exercise influence over events that affect their lives [32]. Optimism is the belief that one will experience good outcomes in life [32].

### Data collection tools and procedures

A structured self-administered questionnaire was used for data collection by adapting from reviewing pieces of literature [32, 34]. The data collection tool was first prepared in English and then translated to Amharic and back to English to ensure consistency. It has 5 parts: sociodemographic characteristics, work engagement, job resources, personal resources, and job demand section. Six diploma and two BSc degree graduated health professionals were recruited for data collection and supervision, respectively. The principal investigator and supervisors checked the filled questionnaire and gave feedback on daily basis. A day of training was given to supervisors and data collectors about the basic techniques of data collection, data collection tools, processes, and the role of each data collector. Pretest was done on 60 health professionals at unselected health facilities of the Gidi Benchi and Gura Ferda districts of the Benchi-Sheko zone. A necessary modification was made based on the pretest findings, mainly on the sequence of questions, language comprehension, and duration of time taken for filling out the questionnaire. Cronbach’s alpha test was done to check the reliability of each composite variable and its values were in the acceptable range with a minimum value of 0.65 (Supplementary file 1).

### Data management and analysis

Data were entered into Epi-info Version 7 and exported to STATA Version 14 for analysis. Double entry was done to verify the coding, completeness, and consistency of the data. Negatively worded items were reverse scored. Mean, standard deviation (SD), and descriptive and summary tables were used to present the findings. The mean with SD for supervisor support, coworker support, role clarity, reward, resilience, self-efficacy, optimism, emotional demand, cognitive demand, and the workload was calculated by summing up the scores of each item and generating a variable for each dimension. We calculated the mean with the SD of each dimension (Supplementary file 2) and a linear regression model was fitted to identify factors associated with work engagement. Variables with p-value < 0.2 during simple linear regression were the candidates for the multiple linear regression model. We checked the assumptions of multiple linear regression, including normality, multicollinearity, homoscedasticity, and linearity using histogram, variance inflation factors (VIF), het-test, residual, and scatter plots, respectively. The assumption of normality was checked using a histogram. Homoscedasticity was checked using the het-test and all results supported the assumption of homoscedasticity. In addition, we checked the linearity assumption using a scatter plot of the standardized residuals versus the predicted values. In addition, we checked the multicollinearity assumptions using the variance inflation factor (VIF) and the value of all variables was less than 10. Unstandardized β-coefficient with 95% CI and p-value < 0.05 were used to declare factors associated with work engagement.

## Results

### Socio-demographic characteristics of the respondents

A total of 578 health professionals participated in the study with a response rate of 95.5%. Half (51.7%) of the respondents were male and 42.2% of the participants were Orthodox Christians. Nearly two-thirds (61.2%) of participants were married. The mean age and work experience of the participants were 28.8 years (± 4.05 SD) and 4.6 years (± 3.29 SD), respectively. More than half (53.5%) and (53.8%) of the participants were nurses and diploma-graduated health professionals, respectively. Most (87%) and a half (50.3%) of the participants were urban residents working at the hospital, respectively. The mean salary of respondents was ETB 4,474.9 ± (1060.3 SD) and ranged from ETB 3,000 to 10, 106 (Table 1).

**Table 1:** Socio-demographic characteristics of health professionals working in public health facilities of Bench-Sheko zone, 2021 (n = 578)

Variables	Categories	Frequency	Percent (%)
Sex	Male	299	51.7
	Female	279	48.3
Religion	Orthodox	245	42.4
	Protestant	235	40.7
	Muslim	76	13.1
	Others	22	3.8
Current marital status	Married	354	61.2
	Unmarried	224	38.8
Specialization	Nurse	309	53.5
	Midwife	77	13.3
	Public health	66	11.4
	Laboratory	60	10.4
	Pharmacist	40	6.9
	MD & specialties	22	3.8
	Others	4	0.7
Educational status	Diploma	311	53.8
	Degree or above	267	46.2
Experiences	≤ 5 years	394	68.2
	≥ 6 years	166	31.8
Type of facility	Hospital	291	50.3
	Health center	287	49.7
Residence	Urban	503	87.0
	Rural	75	13.0
Current position	Non-managerial	493	85.3
	Managerial	85	14.7
Age in years	20–24	59	10.2
	25–29	296	51.2
	30–34	176	30.5
	> 35	47	8.1
Monthly salary (ETB)	≤ 4,000	233	40.3
	4,001–6,000	305	52.7
	≥ 6,001	40	7.0

### Job demand, job, and personal resources

Concerning job resources, the highest mean score percentage of participants was role clarity, which was 76.6% with a mean of 3.30 (± 0.61SD) while the lowest was perceived reward with a mean score percentage of 51.3% and mean of 2.54 (± 0.89 SD). The highest and lowest mean score percentages were self-efficacy and resilience with personal resource, which was 72.7% and 54.2% with a mean of 3.91 (± 0.79SD) and 3.17 (± 0.60SD), respectively. The highest and lowest mean score percentages among participants about their job demands were cognitive and emotional demands with 66.2% with a mean of 3.65(± 0.78SD) and 50.5% with 3.02 (± 0.95SD), respectively (Table 2).

**Table 2:** Job demand, job and personal resources of health professionals working in public health facilities of Bench-Sheko zone, 2021 (n = 578)

Characteristics	Dimensions	Mean	SD	%SM
Job Resources	Supervisor support	3.45	0.85	61.2
	Coworker support	3.60	0.93	65.0
	Role clarity	3.30	0.61	76.6
	Reward	2.54	0.89	51.3
Personal resource	Resilience	3.17	0.60	54.2
	Self-efficacy	3.91	0.79	72.7
	Optimism	3.84	1.01	71.0
Job demand	Emotional demand	3.02	0.95	50.5
	Cognitive demand	3.65	0.78	66.2
	Workload	3.52	0.85	63.0

**Notes**: % SM = standardized percentage of scale mean score; SD: Standard deviation

### Work engagement

The overall mean score percentage for work engagement among healthcare professionals was 71.7% with a mean of 3.87 (± 0.77 SD). The proportion of participants who scored above the mean of overall work engagement was 64.9%. The mean score percentage for dedication, vigor, and absorption of health professionals was 73.7% with a mean of 3.95 (± 0.76SD); 72.5% with a mean of 3.90 (± 0.80SD), and 69.0% with a mean of 3.76 (± 1.01SD), respectively. The proportion of participants who scored above the mean of professionals’ dedication, vigor, and absorption was 63.1%, 61.4%, and 61.6%, respectively.

Concerning dedication, health professionals’ highest mean score percentage was about the pride in the work they did, but the lowest score was observed about the challenge of their job. Regarding vigor, the highest mean score percentage was reported about participants feeling to go to their work when they get up in the morning, but the lowest score was observed about bursting with energy at their work. Regarding absorption, the highest mean score percentage was reported about healthcare providers feeling happy when they worked intensely, but the lowest score was observed about the difficulty to detach themselves from their job (Table 3).

**Table 3:** Work engagement of health professionals working in public health facilities of Bench-Sheko zone, 2021 (n = 578)

Variables	SD	D	N	A	SA	M(SD)	%SM	Proportion above, mean (%)
Vigor						3.90 ± 0.80	72.5	61.4
I feel bursting with energy at my work	20	89	89	283	97	3.60 ± 1.04	65.0	
I feel strong and vigorous at my job	17	81	48	280	152	3.81 ± 1.06	70.2	
I feel to go to work when I get up in the morning	13	40	28	280	217	4.12 ± 0.94	78.0	
I can continue working for long periods at a time	14	105	30	257	172	3.80 ± 1.12	70.0	
I am very strong mentally at my job	8	58	50	212	250	4.10 ± 1.01	77.5	
I always persevere even when things do not go well	8	67	33	289	181	3.98 ± 0.98	74.5	
Dedication						3.95 ± 0.76	73.7	63.1
I find the work that I do meaningfully and purpose	8	60	18	250	242	4.13 ± 0.98	78.2	
I am enthusiastic about my job	11	47	22	212	286	4.23 ± 0.99	80.7	
My job inspires me	18	49	37	196	278	4.15 ± 1.07	78.7	
I am proud of the work that I do	16	40	19	208	295	4.25 ± 1.00	81.2	
My job is challenging for me	112	143	66	148	109	3.00 ± 1.42	50.0	
Absorption						3.76 ± 1.01	69.0	61.6
Time flies when I am working	22	104	30	214	208	3.83 ± 1.20	70.7	
I forget everything when I am working	28	135	66	166	183	3.59 ± 1.30	64.7	
I feel happy when I am working intensely	14	77	28	225	234	4.01 ± 1.10	75.2	
I am immersed in my work	15	110	38	201	218	3.86 ± 1.19	71.5	
I got carried away when I am working	9	127	44	209	189	3.76 ± 1.17	69.0	
It is difficult to detach myself from my job	39	147	51	173	168	3.50 ± 1.32	62.5	
Overall work engagement						3.87 ± 0.77	71.7	64.9

**Notes**: SD: Strongly disagree; D: Disagree; N: Neutral; A: Agree; SA: Strongly Agree; M (SD): Mean with Standard Deviation; %SM: standardized percentage of mean score

### Factors associated with work engagement

The final model explains 69% (adjusted R Square = 0.69) of the variability in the work engagement of health professionals. We conducted simple linear regression for 20 variables (Supplementary file 3) and those 17 variables that had a p-value of less than 0.2 were fitted into a multiple linear regression model. Then, those variables were subjected to multiple linear regressions to control the confounding effect. Finally, married participants (β = 0.10; 95% CI: 0.005, 0.17); working at health centers (β = 0.31; 95% CI: 0.22, 0.40); co-worker support (β = 0.06; 95% CI: 0.004, 0.11); role clarity (β = 0.14; 95% CI: 0.07, 0.21); reward (β = 0.10 95% CI: 0.05, 0.15); resilience (β = 0.14; 95% CI: 0.07, 0.21); self-efficacy (β = 0.24 95% CI: 0.16, 0.31) and optimism (β = 0.20; 95% CI: 0.15, 0.26) were positively associated with health professionals’ work engagement. On the contrary, cognitive demand (β=-0.06; 95% CI: -0.11, -0.01) was negatively associated with health professionals’ work engagement (Table 4).

**Table 4:** Factors associated with work engagement of health professionals working in public health facilities of Bench-Sheko zone, Ethiopia, 2021(n = 578)

Variables	Unstandardized Coeff (β)	Standardized Coeff (β)	95% CI for β		P-value
			**Lower**	Upper	
Educational status					
Diploma	Ref	Ref			
Degree or above	-0.06	-0.04	-0.15	0.02	0.133
Type of facility					
Hospital	Ref	Ref			
Health centers	0.31	0.20	0.22	0.40	0.001*
Sex					
Male	Ref	Ref			
Female	0.04	0.03	-0.04	0.11	0.278
Current marital status					
Unmarried	Ref	Ref			
Married	0.10	0.05	0.005	0.17	0.037*
Age	0.004	0.02	-0.09	0.02	0.523
Experience	0.01	0.04	-0.005	0.02	0.225
Current position					
Manager	Ref	Ref			
Non-manager	0.0003	0.0001	-0.10	0.10	0.995
Supervisor support	0.03	0.04	-0.02	0.09	0.210
Co-worker support	0.06	0.07	0.004	0.11	0.036*
Role clarity	0.14	0.11	0.07	0.21	0.001*
Reward	0.10	0.12	0.05	0.15	0.001*
Resilience	0.14	0.10	0.07	0.21	0.001*
Self-efficacy	0.24	0.24	0.16	0.30	0.001*
Optimism	0.20	0.27	0.15	0.26	0.001*
Cognitive demand	-0.06	-0.07	-0.11	-0.01	0.011*
Emotional demand	-0.008	-0.01	-0.06	0.04	0.748
Workload	-0.004	-0.004	-0.06	0.05	0.880

Notes: * Significant at p value < 0.05; CI = Confidence interval

## Discussion

We assessed work engagement and contributing factors among health professionals working in Bench-Sheko zone public health facilities, in southwest Ethiopia. In this study, the overall percentage mean score of work engagement was (71.8% [95%CI: 70.4, 73.4]). This finding was in line with a study conducted in Egypt (73.3%) [35].

On the contrary, our finding was lower than studies done in Finland (83%) and South Africa (81%) [36, 37]. The possible justification for the variation in Finland could be due to the differences in study participants that is most of the participants in Finland was old aged and had also managerial position. The discrepancy with South Africa might be due to methodological differences, such as the participants in South Africa were selected using a purposive sampling technique, which might enhance the level of their work engagement. On the contrary, our finding was higher than a study finding from China (59%) [26]. The possible justification for this variation could be the participants in the case of China were only nurses, and they might have had a high workload.

Married health professionals had higher work engagement than that unmarried health professionals. This finding was consistent with the findings from Saudi Arabia [38]. The possible reasons for this might be due to married participants might have stable emotions and could get emotional support from their partner which could reduce the work stress and pressure from job demands. Health professionals working in health centers had better work engagement than those working in hospitals. This might be due to most of the health centers having a low patient flow and this, in turn, contributed to health workers having a low workload. This was supported by the job demand resource (JD-R) model in which workload was considered as one of the job demands and negatively associated with work engagement [39].

In this study, job and personal resources were positively associated with work engagement, and job demand was negatively associated with work engagement. This finding was supported by the JD-R model in which job and personal resources initiated a motivational process that led to work engagement while job demand initiated a health impairment process that led to negative health- outcomes [40–42]. Our findings indicated that co-worker support was a positive predictor of work engagement. This finding was in line with the findings in Belgium and Australia [43, 44]. This might be due to co-worker providing information, feedback, guidance, advice, and encouragement to grow skill that influences the motivational process to enhance their work engagement. Co-worker support decreased the role conflicts and workload which in turn improved work engagement [45].

Participants who had role clarity had higher work engagement. This finding was supported by a study conducted in Australia [46]. The possible justification for this might be due to employees who know what is anticipated of them in their job role had an essential certainty and diligence to handle challenges and work at an ideal level [47]. In our study, healthcare providers who received a certain kind of reward had higher work engagement compared with those who did not receive any positive reward. This finding was agreed with the studies in Japan and a systematic review report on work engagement [14, 24]. This might be due to positive rewards that can motivate employees to work hard to improve their performance and to maintain their right practices. Resilient participants had better work engagement compared with their counterparts. This finding was supported by studies conducted in Germany and China [48, 49]. This might be due to resilience assisting employees in improving their ability to cope with, adapt to, and recover from stress at work and might result in an engagement at work.

Work engagement was higher among participants who had higher self-efficacy. This finding was in line with the findings in Spain and Germany [48, 50]. This might be due to participants who had self-efficacy helping to act and decide autonomously to meet various job demands when they faced their job-related challenges [51]. Participants who had higher optimism were engaged in their work. This finding was supported by a study conducted in Germany [48]. This might be due to optimistic employees directing their energy to attain their objectives because they are naturally positive to assess situations during hardships and tribulations instead of resigning and this might in turn improve their engagement.

In this study, healthcare professionals with higher cognitive demands had a poor engagement in their work. This finding was supported by a study conducted in the Netherlands [52]. This might be due to professionals who had high cognitive demand may have job-related stress and this can in turn reduce employees’ job satisfaction and work engagement. On the contrary, our finding was contradicted by the review reports on compassion fatigue and burnout among healthcare professionals [53]. This might be due to emotionally stable persons being more suitable to implement coping strategies to manage workplace stressors.

### Limitations of the study

Information bias can be introduced associated with the self-reporting of health professionals on their level of work engagement. In addition, our study lacks qualitative findings to explore non-quantifiable factors that might contribute to professionals’ work engagement.

## Conclusion

The overall work engagement of health professionals in the Bench-Sheko zone was judged as average according to the UWES manual. Health professionals working at health centers, married professionals, co-worker support, role clarity, reward, resilience, self-efficacy, optimism, and cognitive demand were significantly associated with work engagement. Health facilities shall improve their culture of co-worker support, role clarity, reward, resilience, self-efficacy, and optimism to enhance work engagement. Future researchers shall be done further studies to evaluate the relationship between cognitive demand and work engagement among health professionals. In addition, policymakers and health managers shall take into consideration rewarding mechanisms and promote the self-efficacy of employees to enhance work engagement. The authors of this study recommended future researchers conduct a mixed methods study to contribute sufficient evidence for policymakers.

## Electronic supplementary material

Below is the link to the electronic supplementary material.

Supplementary Material 1

Supplementary Material 2

Supplementary Material 3

## Data Availability

Data will be available upon reasonable request from the corresponding author.

## Abbreviations

BRS: Brief Resilience Scale
COPSOQ: Copenhagen Psychosocial Questionnaire
HCO: Health Care Organizations
HC: Health Center
HW: Health Worker
HWF: Health Work Force
IRB: Institutional Review Board
JD-R: Job Demand-Resource
MTUTH: Mizan Tepi University Teaching Hospital
PHF: Public Health Facilities
SNNPR: Southern Nations, Nationalities, and People’s Region
SDG: Sustainable Development Goals
SPSS: Statistical Package for Social Sciences
SSA: Sub-Saharan Africa
SWE: Southwest Ethiopia
UHC: Universal Health Coverage
UWES: Utrecht Work Engagement Scale
