# Supplementary material for: Work engagement among health professionals in public health facilities of Bench-Sheko zone, southwest Ethiopia
Source: BMC Health Serv Res. 2023 Jun 27;23:697. doi: 10.1186/s12913-023-09680-5 (PMC10294362; doi:10.1186/s12913-023-09680-5)
Supplement: Supplementary file 1 — Supplementary Material 1 [file 12913_2023_9680_MOESM1_ESM.docx]

Table S1: Cronbach’s alpha values

| Characteristics | Number of items | Likert scale | Cronbach’s alpha |
| --- | --- | --- | --- |
| Vigor | Six | 1: strongly disagree to 5: strongly agree | 0.87 |
| Dedication | Five | 1: strongly disagree to 5: strongly agree | 0.77 |
| Absorption | Six | 1: strongly disagree to 5: strongly agree | 0.91 |
| Supervisor support | Three | 1: never/hardly ever, 2: seldom, 3: sometimes, 4: often, and 5: always | 0.81 |
| Coworker support | Three | 1: never/hardly ever, 2: seldom, 3: sometimes, 4: often, and 5: always | 0.86 |
| Cognitive demand | Four | 1: never/hardly ever, 2: seldom, 3: sometimes, 4: often, and 5: always | 0.75 |
| Emotional demand | Four | 1: never/hardly ever, 2: seldom, 3: sometimes, 4: often, and 5: always | 0.79 |
| Role clarity | Three | 1: very small extent, 2: small extent, 3: large extent, 4: very large extent | 0.83 |
| Reward | Three | 1: very small extent, 2: small extent, 3: large extent, 4: very large extent | 0.87 |
| Resilience | Six | 1: strongly disagree to 5: strongly agree | 0.65 |
| Workload | Seven | 1: strongly disagree to 5: strongly agree | 0.86 |
| Self-efficacy | Six | 1: strongly disagree to 5: strongly agree | 0.91 |
| Optimism | Three | 1: strongly disagree to 5: strongly agree | 0.86 |
