# Supplementary material for: Work engagement among health professionals in public health facilities of Bench-Sheko zone, southwest Ethiopia
Source: BMC Health Serv Res. 2023 Jun 27;23:697. doi: 10.1186/s12913-023-09680-5 (PMC10294362; doi:10.1186/s12913-023-09680-5)
Supplement: Supplementary file 2 — Supplementary Material 2 [file 12913_2023_9680_MOESM2_ESM.docx]

| **Statistics** | | | | | | | | | | | |
| --- | --- | --- | --- | --- | --- | --- | --- | --- | --- | --- | --- |
|  | | supervisor_support | coworker_support | role_clarity | reward | Resilience | self_efficacy | optimism | emotional_demand | cognitive_demand | workload |
| N | Valid | 578 | 578 | 578 | 578 | 578 | 578 | 578 | 578 | 578 | 578 |
|  | Missing | 0 | 0 | 0 | 0 | 0 | 0 | 0 | 0 | 0 | 0 |
| Mean | | 3.44982698961938 | 3.60034602076125 | 3.30334486735871 | 2.54152249134948 | 3.17935409457901 | 3.91608996539792 | 3.84659746251442 | 3.0208 | 3.6488 | 3.52125556104795 |
| Std. Deviation | | .855359384886738 | .927583944052783 | .615500685334163 | .891530115490335 | .597214574003219 | .795365362623297 | 1.013065500867563 | .94715 | .87605 | .856434967079578 |
