# Supplementary material for: Work engagement among health professionals in public health facilities of Bench-Sheko zone, southwest Ethiopia
Source: BMC Health Serv Res. 2023 Jun 27;23:697. doi: 10.1186/s12913-023-09680-5 (PMC10294362; doi:10.1186/s12913-023-09680-5)
Supplement: Supplementary file 3 — Supplementary Material 3 [file 12913_2023_9680_MOESM3_ESM.docx]

| Variables | Unstandardized Coeff (β) | Standardized  Coeff (β) | 95% CI for unstandardized β | | P-value |
| --- | --- | --- | --- | --- | --- |
|  |  |  | Lower | Upper |  |
| Educational status |  |  |  |  |  |
| Diploma | Ref | Ref |  |  |  |
| Degree or above | -0.42 | -0.27 | -0.54 | -0.30 | < 0.001 |
| Type of facility |  |  |  |  |  |
| Hospital | Ref | Ref |  |  |  |
| Health centers | 0.45 | 0.29 | 0.33 | 0.57 | < 0.001 |
| Sex |  |  |  |  |  |
| Male | Ref | Ref |  |  |  |
| Female | 0.09 | 0.06 | -0.04 | 0.22 | 0.16 |
| Current marital status |  |  |  |  |  |
| Unmarried | Ref | Ref |  |  |  |
| Married | -0.43 | -0.27 | -0.55 | -0.30 | < 0.001 |
| Age | 0.04 | 0.21 | 0.03 | 0.06 | < 0.001 |
| Experience | 0.06 | 0.24 | 0.04 | 0.07 | < 0.001 |
| Current position |  |  |  |  |  |
| Manager | Ref | Ref |  |  |  |
| Non-manager | -0.22 | -0.10 | -0.40 | -0.05 | 0.014 |
| Supervisor support | 0.45 | 0.50 | 0.39 | 0.51 | < 0.001 |
| Co-worker support | 0.49 | 0.56 | 0.41 | 0.52 | < 0.001 |
| Role clarity | 0.70 | 0.56 | 0.61 | 0.79 | < 0.001 |
| Reward | 0.44 | 0.50 | 0.38 | 0.49 | < 0.001 |
| Resilience | 0.56 | 0.44 | 0.47 | 0.66 | < 0.001 |
| Self-efficacy | 0.70 | 0.72 | 0.65 | 0.76 | < 0.001 |
| Optimism | 0.49 | 0.64 | 0.44 | 0.53 | < 0.001 |
| Cognitive demand | -0.22 | -0.25 | -0.29 | -0.15 | < 0.001 |
| Emotional demand | -0.28 | -0.34 | -0.34 | -0.21 | < 0.001 |
| Workload | -0.33 | -0.36 | -0.40 | -0.26 | < 0.001 |
| Residence |  |  |  |  |  |
| Rural | Ref | Ref |  |  |  |
| Urban | -0.08 | -0.04 | -0.27 | 0.11 | 0.40 |
| Monthly salary | -0.11 | -0.05 | -0.29 | -0.07 | 0.21 |
| Profession |  |  |  |  |  |
| Other professionals | Ref | Ref |  |  |  |
| Nurse or midwifery | -0.03 | -0.02 | -0.16 | 0.11 | 0.68 |

Table S2: Simple linear regression for each of independent variables with work engagement
